# Supplementary material for: Glutaminases as a Novel Target for SDHB-Associated Pheochromocytomas/Paragangliomas
Source: Cancers (Basel). 2020 Mar 5;12(3):599. doi: 10.3390/cancers12030599 (PMC7139890; doi:10.3390/cancers12030599)
Supplement: Supplementary file 1 [file cancers-12-00599-s001.pdf]

Supplementary Materials: Glutaminases as a Novel Target for SDHB-Associated Pheochromocytomas/Paragangliomas

Balazs Sarkadi, Katalin Meszaros, Ildiko Krencz, Letizia Canu, Lilla Krokker, Sara Zakarias, Gabor Barna, Anna Sebestyen, Judit Papay, Zoltan Hujber, Henriett Butz, Otto Darvasi, Peter Igaz, Judit Doczi, Michaela Luconi, Christos Chinopoulos and Attila Patocs

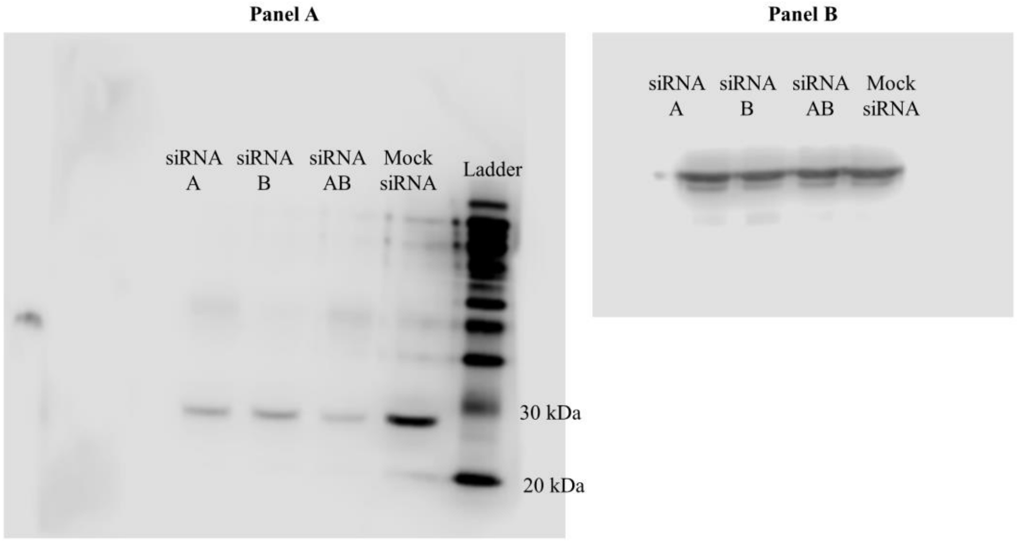

Figure S1. *SDHB* knockdown by *Sdhb* targeting siRNAs. Two different *SDHB* targeting siRNAs (see materials and methods) were used for *SDHB* knockdown, labeled as siRNA A and siRNA B. The combination of two siRNAs was labeled as siRNA AB (which is named as 'si*Sdhb*' in the manuscript). Panel **A**: Sdhb targeted Western blot membrane after transfection with siRNA A, siRNA B, mock siRNA or the combination of siRNA A and B. Panel **B**: β-actin targeted Western blot membrane after transfection with siRNA A, siRNA B, mock siRNA or the combination of siRNA A and B.

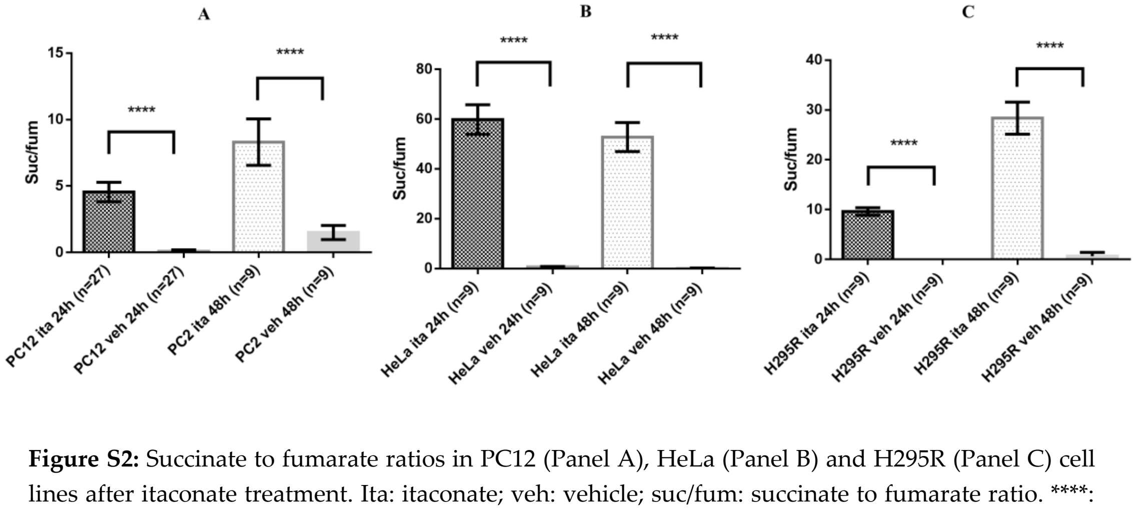

Figure S2: Succinate to fumarate ratios in PC12 (Panel A), HeLa (Panel B) and H295R (Panel C) cell lines after itaconate treatment. Ita: itaconate; veh: vehicle; suc/fum: succinate to fumarate ratio. ****: $p < 0.0001$.

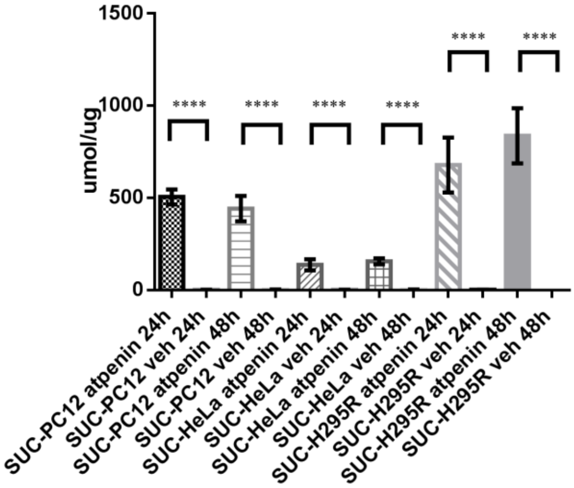

Figure S3: Normalized succinate levels after atpenin treatment in PC12, HeLa and H295R cell lines. veh: vehicle; suc: succinate. ****: $p < 0.0001$.

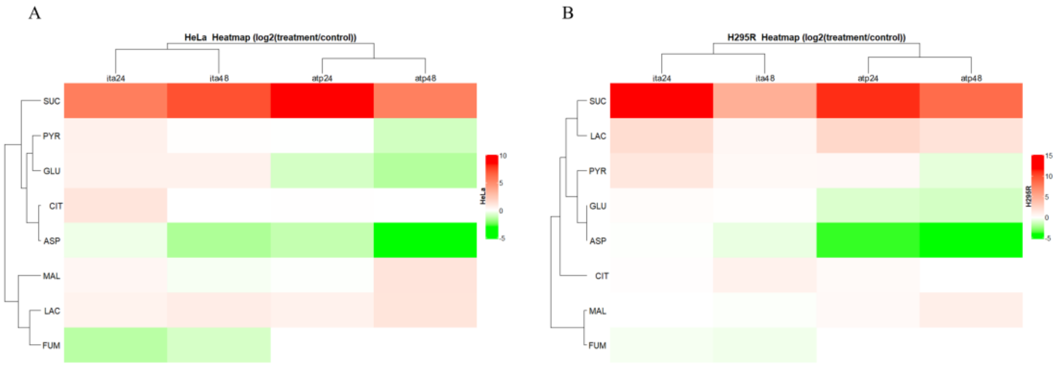

Figure S4. HeatMap. Visualization of changes in metabolite concentrations in HeLa (Panel **A**) and H295R (Panel **B**) cell lines after itaconate and atpenin treatment. Fold-changes of different metabolic concentration were calculated (values measured after treatment were divided with control values) then the given values were log2 transformed. These values were used for construction of the heatmap and represented with color scale (red+/white 0/green−). atp24: 24 h atpenin treatment; atp48: 48 h atpenin treatment; ita24: 24 h itaconate treatment; ita48: 48 h itaconate treatment; SUC: succinate; LAC: lactate; PYR: pyruvate; CIT: citrate; MAL: malate; FUM: fumarate; GLU: glutamate; ASP: aspartate.

Table 1. Intracellular normalized concentrations of all measured metabolites (μmol/μg). All data are presented as mean ± SD. CIT: citrate; FUM: fumarate; LAC: lactate; MAL: malate; PYR: pyruvate; SUC: succinate; ASP: aspartate; GLU: glutamate. Ita: itaconate treatment; veh: vehicle treatment. NA: not applicable.

Samples	FUM	*p*	LAC	*p*	MAL	*p*	PYR	*p*	SUC	*p*	ASP	*p*	GLU	*p*	CIT	*p*
PC12 ita 24 h	7.9 ± 1.1	< 0.0001	278.1 ± 58.6	0.4705	20.2 ± 2.5	0.9034	109.4 ± 41	0.2469	35.2 ± 3.8	< 0.0001	209.3 ± 27.3	0.4267	849.8 ± 74.2	0.008	31.16 ± 2.2	< 0.0001
PC12 veh 24 h	9.9 ± 1		264.3 ± 66.6		20.1 ± 2.1		88.9 ± 24.8		0.9 ± 1.1		207.9 ± 16.2		895. 2 ± 40.6		33.75 ± 2.2	
PC12 ita 48 h	2.1 ± 0.7	0.7452	295.1 ± 112.9	0.8954	0	NA	109.6 ± 39.2	0.3496	16.7 ± 3.5	< 0.0001	85.3 ± 19.9	0.2474	455.1 ± 90.7	0.5387	55.4 ± 13.6	0.077
PC12 veh 48 h	2 ± 0.5		287.4 ± 130.2		0		91.7 ± 39.4		2.8 ± 0.4		94.2 ± 9.2		474.8 ± 23.9		41.4 ± 2.2	
PC12 si*Sdhb*	2.8 ± 0.7	0.0013	217.4 ± 100	0.827	33.9 ± 10.1	0.0269	45.2 ± 17.9	0.7137	2.8 ± 0.4	< 0.0001	122.5 ± 26.7	< 0.0001	496.5 ± 110.2	< 0.0001	0	NA
PC12 mock siRNA	3.7 ± 0.1		238.4 ± 70.5		43.1 ± 3.43		50.5 ± 11.9		1.2 ± 0.4		208.3 ± 31.2		743.5 ± 71.7		0	
HeLa ita 24 h	2.8 ± 0.8	< 0.0001	355.8 ± 106.5	0.0135	46 ± 5.9	0.0188	13.6 ± 2.8	< 0.0001	169 ± 49.8	< 0.0001	35 ± 7.2	< 0.0001	489.7 ± 113.5	0.0471	21.5 ± 2.7	< 0.0001
HeLa veh 24 h	6.9 ± 0.7		243.7 ± 24.6		35.2 ± 8		8.8 ± 1.5		3.5 ± 0.7		44.1 ± 15.2		328 ± 63.7		9.6 ± 7.2	
HeLa ita 48 h	2.5 ± 0.3	< 0.0001	1517.5 ± 835.4	0.0181	23.2 ± 5.5	0.5309	12.3 ± 3.7	0.7298	130.9 ± 24.2	< 0.0001	16.2 ± 2.8	< 0.0001	496.3 ± 49.5	< 0.0001	0	NA
HeLa veh 48 h	4.4 ± 0.7		877.9 ± 90.3		26.2 ± 3.7		11.8 ± 1.6		0.9 ± 0.08		45.4 ± 4.1		328.2 ± 34.3		0	
H295R ita 24 h	2.5 ± 0.3	0.0014	110.6 ± 36.4	< 0.0001	0	NA	52 ± 14.5	< 0.0001	24.2 ± 2.7	< 0.0001	92 ± 17.3	0.2547	174.5 ± 24.5	0.0188	41.4 ± 4.6	0.1020
H295R veh 24 h	3 ± 0.3		26.4 ± 6.5		0		18.5 ± 2.3		0.007 ± 0.02		96 ± 7		151.2 ± 9.6		38.5 ± 2.2	
H295R ita 48 h	2.7 ± 0.4	0.0012	278 ± 115.9	0.1883	38.1 ± 6	0.7137	24.5 ± 9.9	0.1421	76.9 ± 11	< 0.0001	67.9 ± 8.8	< 0.0001	309.4 ± 41.4	0.4799	13 ± 1	< 0.0001
H295R veh 48 h	3.6 ± 0.5		201.5 ± 91.4		40.8 ± 8.8		18.6 ± 5.5		2.65 ± 3.38		101.7 ± 12.3		294.8 ± 35.4		7.5 ± 5.7	

Table S2. Intracellular normalized concentrations of all measured metabolites (µmol/µg). All data are presented as mean ± SD. CIT: citrate; FUM: fumarate; LAC: lactate; MAL: malate; PYR: pyruvate; SUC: succinate; ASP: aspartate; GLU: glutamate. Ita: itaconate treatment; veh: vehicle treatment.

Samples	FUM	*p*	LAC	*p*	MAL	*p*	PYR	*p*	SUC	*p*	ASP	*p*	GLU	*p*	CIT	*p*
PC12 atpenin 24 h	1.3 ± 0.5	<0.0001	198.3 ± 58	0.004	19 ± 0.9	<0.0001	66 ± 18	0.9132	504.4 ± 40.7	<0.0001	42.6 ± 2.9	<0.0001	334.8 ± 12.7	<0.0001	15.1 ± 0.1	<0.0001
PC12 veh 24 h	5.6 ± 0.6		122.1 ± 48		22.2 ± 0.9		61.4 ± 19		0.4 ± 0.9		139.1 ± 11.4		582.1 ± 36.1		15.3 ± 0.0	
PC12 atpenin 48 h	0	<0.0001	332.2 ± 240.2	< 0.0001	14 ± 2	<0.0001	51.6 ± 36.4	0.0229	441 ± 68.4	<0.0001	18.4 ± 4.5	<0.0001	138.7 ± 18.5	<0.0001	10.2 ± 1.1	<0.0001
PC12 veh 48 h	1.7 ± 0.2		67 ± 36.8		10 ± 0.3		20.7 ± 10.1		1.3 ± 0.3		50.6 ± 1.3		244.6 ± 9		7.4 ± 0.3	
HeLa atpenin 24 h	0	<0.0001	297.7 ± 33.3	0.0039	16 ± 2.4	0.5962	12.6 ± 1.7	0.9073	136 ± 29.5	<0.0001	6.8 ± 1.3	<0.0001	51.8 ± 10.2	<0.0001	10.4 ± 1.2	0.7142
HeLa veh 24 h	1.9 ± 0.3		197.7 ± 77.5		16.5 ± 1.8		12.7 ± 4.3		0.4 ± 0.6		14.8 ± 2		93.1 ± 11.2		10.2 ± 1.1	
HeLa atpenin 48 h	0	<0.0001	785.2 ± 188.4	< 0.0001	35.8 ± 4.2	<0.0001	4.5 ± 1.5	0.0003	155.3 ± 16.2	<0.0001	2.8 ± 0.4	<0.0001	85 ± 8.7	<0.0001	0	NA
HeLa veh 48 h	5.1 ± 0.8		785.2 ± 188.4		15.7 ± 1.7		8.3 ± 1.9		2 ± 0.7		20.6 ± 4		224.5 ± 40.7		0	
H295R atpenin 24 h	0	<0.0001	372.7 ± 166.4	< 0.0001	35.5 ± 1.5	<0.0001	23.8 ± 6.7	0.0229	678 ± 148.7	<0.0001	7.4 ± 0.7	<0.0001	83.5 ± 3.4	<0.0001	22.2. ± 1.3	<0.0001
H295R veh 24 h	3.5 ± 0.7		70.9 ± 56.8		28.1 ± 2.8		29.5 ± 14.3		0.3 ± 0.9		100.7 ± 20		165.2 ± 30.7		17.8 ± 1.3	
H295R atpenin 48 h	0	<0.0001	1473.1 ± 761.1	0.004	73.9 ± 10.6	<0.0001	29.5 ± 14.3	0.0767	836 ± 150	<0.0001	5.5 ± 1.1	<0.0001	122.8 ± 15.4	<0.0001	0	NA
H295R veh 48 h	3.8 ± 0.7		454.5 ± 236.8		37.9 ± 1.4		48.1 ± 26.8		0		85 ± 9.9		277.1 ± 18.3		0	

© 2020 by the authors. Licensee MDPI, Basel, Switzerland. This article is an open access article distributed under the terms and conditions of the Creative Commons Attribution (CC BY) license (http://creativecommons.org/licenses/by/4.0/).
